# Targeting the inhibitors of apoptosis proteins (IAPs) to combat drug resistance in cancers

**DOI:** 10.3389/fphar.2025.1562167

**Published:** 2025-03-28

**Authors:** Qingmei Ye, Xiao-Zhao Zhuang, Juan Li, Xin Zhou

**Affiliations:** ^1^ Hainan General Hospital and Hainan Affiliated Hospital of Hainan Medical University, Haikou, Hainan, China; ^2^ Hubei Province Key Laboratory of Traditional Chinese Medicine Resource and Chemistry, Department of Pharmacy, Hubei University of Chinese Medicine, Wuhan, Hubei, China; ^3^ The Fifth People’s Hospital of Hainan Province and Affiliated Dermatology Hospital of Hainan Medical University, Haikou, Hainan, China

**Keywords:** drug resistance, cancer, inhibitors of apoptosis proteins, inhibitors, overcome

## Abstract

Inhibitors of Apoptosis Proteins (IAPs) are a family of anti-apoptotic proteins that play a pivotal role in apoptosis in general but also as oncoproteins in cancer progression and, more importantly, drug resistance. IAPs enable cancer cells to evade programmed cell death and adapt to therapeutic stress by inhibiting pro-apoptotic caspase activity as well as modulating pivotal survival pathways. Recent advancements in targeting IAPs, particularly through the use of SMAC (second mitochondria-derived activator of caspase) mimetics and other small-molecule antagonists or inhibitors, have opened new avenues for overcoming drug resistance in cancers. The current review attempted to summarize the *status quo* of IAPs’ role in promoting chemotherapeutic drug resistance in various cancer treatments and discuss the most recent development of IAP-targeting therapies, particularly small-molecule inhibitors including their combinational strategies to enhance the sensitivity or achieve synergism to existing therapeutics. Additionally, we also outline the challenges and offer future perspectives for optimizing IAP-targeted approaches to improve clinical outcomes.

## 1 Introduction

Currently, most malignant cancers and tumors have been managed to be controllable and are sometimes regarded as chronic diseases if diagnosed at an early stage and treated in time and effectively. However, cancer therapy often faces a formidable challenge of drug resistance, which leads to treatment failure and poor prognosis for patients (Zhang et al., 2023; Cui et al., 2022). More strikingly, drug resistance may account for roughly 90% of cancer-related deaths, rendering it an urgent issue to address. (Pluchino et al., 2012). Drug resistance usually arises from the ability of cancer cells to evade apoptosis, survive in adverse conditions, and proliferate despite the presence of cytotoxic chemotherapeutic agents or targeted therapies, including those cutting-edge immunotherapies (Narayanan et al., 2020; Su, 2022; Perez-Ruiz et al., 2020). The reasons conferring drug resistance are often complicated and multifaceted, including inactive metabolites by chromosomes, enhanced anti-apoptotic ability, decreased drug concentration inside cancer cells by active membrane transporters, mutation of the target, shielding of the drug to reach the target, inability to completely eliminate cancer cells, including cancer stem cells, etc. (Assaraf et al., 2019; Sajid et al., 2023; Sancho et al., 2016). Of note, one primary factor can often cause resistance to multiple drugs, termed multidrug resistance (MDR). Likewise, multiple factors may also contribute to one drug’s resistance, rendering it a conundrum to tackle.

Among the key players in these processes are the Inhibitors of Apoptosis Proteins (IAPs), which are overwhelmingly overexpressed in almost all cancer types (Ramakrishnan et al., 2014; Shahar and Larisch, 2020; Rathore et al., 2017). Recently, there has been growing interest in targeting IAPs as therapeutic strategies to overcome drug resistance. Theoretically, by inhibiting the proteins that suppress apoptosis, researchers aim to restore the natural cell death processes and sensitize tumors to those existing treatments. This review highlights the information on IAPs in inducing drug resistance in cancers and discusses the potential of targeting IAPs as an effective approach to tackling drug resistance.

## 2 IAPs and their roles in inducing drug resistance in cancers

### 2.1 IAPs function to suppress apoptosis

The human IAP family comprises eight members, including neuronal apoptosis inhibitory protein (NAIP), cellular IAP 1 (c-IAP1), cellular IAP 2 (c-IAP2), X-linked IAP (XIAP), survivin, Baculovirus IAP Repeat (BIR)-containing ubiquitin-conjugating enzyme (BRUCE or Apollon), melanoma IAP (ML-IAP or Livin), and IAP-like Protein 2 (ILP2) (Table 1), which are functioning to apoptosis regulation (Cui et al., 2023; Cetraro et al., 2022). Generally, these proteins inhibit caspase activity, thereby preventing the execution of apoptosis (Cetraro et al., 2022). However, it should be noted that not all eight members have been clearly and fully elucidated in terms of their enzymatic/interactive functions in cancer cells, except XIAP, c-IAP1/2, survivin, and BRUCE, which will be discussed briefly below.

**TABLE 1:** Summary of IAPs conferring drug resistance in different cancers.

IAPs	Chemotherapeutics	Cancer (cell) types	References
NAIP	Not reported		
c-IAP1/2	Doxorubicin	Leukemia	Vaziri et al. (2003)
		Pancreatic cancer	Lopes et al. (2007)
	Taxol	Pancreatic cancer	Lopes et al. (2007)
	Cisplatin	Pancreatic cancer	Lopes et al. (2007)
	Vincristine	Neuroblastoma	Frommann et al. (2018)
XIAP	Taxol	Prostate cancer	Nomura et al. (2003)
		NSCLC	Liu et al. (2011)
		Pancreatic cancer	Lopes et al. (2007)
		Breast cancer	Lin et al. (2004)
	Docetaxel	Prostate cancer	Zhang et al. (2021)
		Ovarian cancer	Sapi et al. (2004)
		Pancreatic cancer	Lopes et al. (2007)
	Doxorubicin	Breast cancer	Delbue et al. (2020), Lin et al. (2004)
	Lapatinib	Breast cancer	Aird et al. (2010)
	Imatinib	CML	Silva et al. (2013)
	Venetoclax	CLL	Takacs et al. (2021)
	Cisplatin	Colon cancer	Xiong et al. (2017)
		Ovarian cancer	Ma et al. (2009), Rada et al. (2018)
		NSCLC, SCLC	Xu et al. (2017), Liu et al. (2011)
		Pancreatic cancer	Lopes et al. (2007)
	Gemcitabine	Pancreatic cancer	Shrikhande et al. (2006)
	Carboplatin	Ovarian cancer	Thibault et al. (2018)
	Vincristine	Neuroblastoma	Frommann et al. (2018)
Survivin	Sorafenib	HCC	Sun et al. (2021), Liu et al. (2023)
	Imatinib	CML	Speletas et al. (2011), Carter et al. (2006)
	Doxorubicin	HCC	Liu et al. (2023)
		Breast cancer	Faversani et al. (2014), Liu et al. (2007)
		AML	Wang et al. (2003)
	Vincristine	Lung cancer	Zhou J. et al. (2018), Park et al. (2011), Tsubaki et al. (2015)
		ALL	Park et al. (2011)
		Myeloma	Tsubaki et al. (2015)
	Docetaxel	Lung cancer	Li et al. (2023)
	Gemcitabine	Pancreatic cancer	Fuller et al. (2022)
	Tamoxifen	Breast cancer	Yu et al. (2020)
	Cisplatin	Bladder cancer	Krafft et al. (2019)
		Gastric cancer	Dong et al. (2014), Sun et al. (2014)
		NSCLC	Hu et al. (2016)
	Gefitinib	Lung cancer	Zhou C. et al. (2018)
	Daunorubicin	ALL	Wu et al. (2008)
	Cytarabine	AML	Stroopinsky et al. (2018)
	Taxol	Ovarian cancer	Zaffaroni et al. (2002)
		NSCLC	Shen et al. (2012)
BRUCE	Doxorubicin	CML	Chen et al. (2017)
	Imatinib	CML	Okumu et al. (2017)
	Temozolomide	Melanoma	Tassi et al. (2012)
Livin	Cisplatin	Colon cancer	Ding et al. (2013)
	Vincristine Etoposide 5-Fluorouracil (5-FU)	Colon cancer	Wang et al. (2010)
ILP2	Not reported		

Note: NSCLC, non-small cell lung cancer; CML, chronic myeloid leukemia; CLL, chronic lymphocytic leukemia; SCLC, small cell lung cancer; HCC, hepatocellular carcinoma; AML, acute myeloid leukemia; ALL, acute lymphoblastic leukemia.

XIAP is the most extensively studied member of IAPs in apoptosis and in cancer, and it can exert its function through direct and indirect mechanisms. XIAP appears to be the only one that potently inhibits the enzymatic activity of the proapoptotic caspases (Kashkar, 2010). Briefly, through a direct interaction, XIAP may bind to and inhibit caspases-3, -7, and -9, thereby inhibiting the initiation and/or the cascade of apoptotic event (Chai et al., 2001; Riedl et al., 2001; Shiozaki et al., 2003). In indirect ways, XIAP may 1) undermine mitochondria-mediated apoptosis, delaying the release of cytochrome *c*, apoptotic protease activating factor 1 (Apaf-1), and second mitochondria-derived activator of caspase (SMAC) with the involvement of Bcl-2 family proteins (Chen et al., 2018; Flanagan et al., 2010); 2) facilitate the ubiquitination and the subsequent degradation of proapoptotic SMAC (Qin et al., 2016; Macfarlane et al., 2002); And 3) interacting with other players, such as microRNA (Xie et al., 2013), HS1-associated protein X1 (HAX-1) (Kang et al., 2010), etc.

c-IAP1 and c-IAP2 are critical regulators of apoptosis, primarily acting through their E3 ubiquitin ligase activity instead of directly inhibiting caspases (Bertrand et al., 2008). c-IAP1/2 modulate key signaling pathways, particularly those involving the tumor necrosis factor receptor 1 (TNFR1) and NF-κB, to maintain cellular homeostasis (Varfolomeev et al., 2008). In the canonical TNFR1 signaling pathway, c-IAP1/2 ubiquitinate receptor-interacting protein kinase 1 (RIPK1) at the TNFR1 signaling complex, promoting the activation of NF-κB, which is a transcription factor that drives the expression of pro-survival and anti-apoptotic genes (Varfolomeev et al., 2008). This ubiquitination prevents the formation of the pro-apoptotic complex IIb, which includes RIPK1, Fas-Associated protein with Death Domain (FADD), and caspase-8, thereby suppressing apoptosis (Varfolomeev et al., 2008; Dynek et al., 2010). In addition to regulating the canonical NF-κB pathway, c-IAP1/2 play a crucial role in the non-canonical NF-κB pathway by targeting NF-κB-inducing kinase (NIK) for continuous ubiquitination and proteasomal degradation (Zarnegar et al., 2008). This regulation keeps NIK levels low under basal conditions, preventing unwanted activation of non-canonical NF-κB signaling (Zarnegar et al., 2008). The genetic depletion or pharmacological inhibition of c-IAP1/2 disrupts their ubiquitin ligase function, leading to the accumulation of RIPK1 in its non-ubiquitinated form (Darding and Meier, 2012). This allows RIPK1 to switch roles, forming pro-apoptotic or pro-necroptotic complexes, depending on the cellular context (Schorn et al., 2023; Darding and Meier, 2012).

One intricate property of IAPs is that the different members may form a complex, e.g., the formation of a survivin-XIAP complex could promote increased XIAP stability against its ubiquitination and proteasomal destruction/degradation and, thus, lead to synergistic inhibition of apoptosis (Dohi et al., 2004). Survivin has been suggested to bind directly to caspases-3 and -7, and then inhibit their activation in proapoptosis (Shin et al., 2001). However, other studies have shown the opposite results since the chemically synthesized survivin failed to inhibit caspase 3 activity (Li et al., 2008), and a previous study also suggested that mouse and human survivin did not target and suppress caspase 3 (Banks et al., 2000). Likewise, survivin was predicted to bind to caspase 9 and inhibit its activation (O'Connor et al., 2000); however, a following study validated that survivin worked cooperatively with hepatitis B X-interacting protein (HBXIP) to bind to pro-caspase 9 and thereby, inhibiting its activation and suppressing apoptosis (Marusawa et al., 2003). While existing naturally as a dimer, monomer survivin also exerts anti-apoptotic effects via directly interacting with SMAC/DIABLO and XIAP (Pavlyukov et al., 2011). As aforementioned, survivin majorly inhibits apoptosis through the formation of a complex with other IAPs or other players in regulating apoptosis pathways. Survivin directly interacts with SMAC/DIABLO in the mitochondria, preventing its release into the cytosol and subsequent activation of apoptosis (Ceballos-Cancino et al., 2007).

BRUCE acts as both a suppressor of apoptosis and an E3 ubiquitin ligase, influencing apoptotic pathways through several mechanisms, including 1) inhibition of caspase activation. BRUCE binds to and inhibits pro-apoptotic SMAC/DIABLO and HtrA2/Omi, which are released from mitochondria during apoptosis, through which BRUCE prevents them from neutralizing XIAP, thereby indirectly suppressing caspase activation (Qiu and Goldberg, 2005). 2) ubiquitination of pro-apoptotic proteins. BRUCE ubiquitinates and targets apoptotic proteins, e.g., caspase 9, for proteasomal degradation, maintaining a balance between pro-survival and pro-death signals (Hao et al., 2004); 3) regulating mitochondrial integrity. BRUCE contributes to maintaining mitochondrial integrity, which is critical for preventing the release of apoptogenic factors such as cytochrome *c* and SMAC/DIABLO (Ren et al., 2005); and 4) regulating the extrinsic apoptotic pathway. BRUCE modulates the extrinsic pathway by controlling the turnover of death receptor-associated proteins, thereby influencing cell death triggered by certain extracellular signals (Liu et al., 2024).

In addition, other IAPs members have also been validated to suppress apoptosis through various mechanisms. NAIP directly inhibits caspases −3 and −7 (Maier et al., 2002), and it could inhibit procaspase-9 (Davoodi et al., 2010; Karimpour et al., 2011). Similar to NAIP, Livin also interacts with caspases-3, -7 and -9 (Kasof and Gomes, 2001). Finally, ILP2 appears to be unstable to successfully inhibit the activation of caspases (Shin et al., 2005).

### 2.2 IAPs induce drug resistance in cancers

**FIGURE 1:**
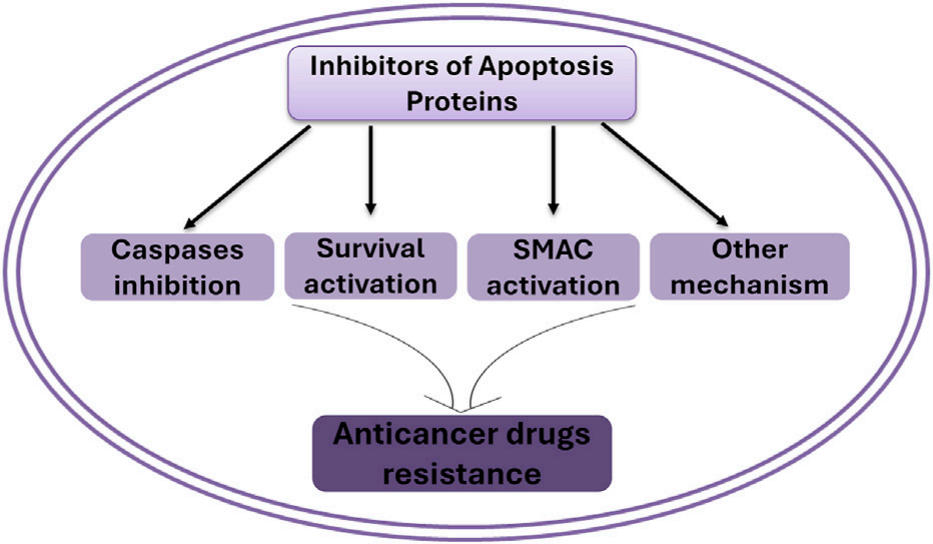
IAPs induces drug resistance via multiple mechanisms.

IAPs are frequently overexpressed in tumors, correlating with poor prognosis, enhanced tumor aggressiveness, and increased resistance to various therapies (Liang et al., 2020). IAPs contribute to drug resistance through several interconnected mechanisms that are similar and consistent with their general biological role, such as 1) apoptosis inhibition. IAPs may bind or interact with caspases, rendering them inactive and halting the apoptotic cascade in response to cytotoxic therapies (Shiozaki et al., 2003; Maier et al., 2002); 2) activation of survival pathways. c-IAP1/2 can activate NF-κB, promoting transcription of genes involved in cell proliferation, and anti-apoptotic processes (Varfolomeev et al., 2008); 3) disruption of SMAC/DIABLO activity. SMAC/DIABLO, an endogenous antagonist of IAPs, is often inactivated or sequestered, modulating the balance toward apoptosis inhibition, which can be reversed by IAPs (Ceballos-Cancino et al., 2007); and 4) coordination with other critical signal pathways to suppress cell death as we discussed above (Figure 1).

In a word, IAPs suppress apoptosis irrespective of the underlying causes, thereby contributing to MDR across a wide range of chemotherapeutic agents. This broad-spectrum resistance poses significant challenges in cancer treatment, necessitating the development of targeted strategies to overcome IAP-mediated MDR and restore the efficacy of conventional therapies.

In Table 1, we summarized the eight members and their associated drug resistance (no study has been published regarding NAIP or ILP2 as the leading/main role in causing drug resistance in cancers). Particularly, we would like to briefly discuss the drug resistance caused by XIAP and survivin, which are the two most studied and play a leading role in conferring drug resistance in cancers (Li et al., 2000).

As shown in Table 1, XIAP contributes to resistance against Taxol in prostate, lung, pancreatic, and breast cancers, emphasizing its involvement in protecting cancer cells from apoptosis induced by this commonly used microtubule-targeting agent (Nomura et al., 2003; Lin et al., 2004; Lopes et al., 2007). XIAP mediates resistance to Docetaxel in prostate, ovarian, and pancreatic cancers (Zhang et al., 2021; Lopes et al., 2007; Sapi et al., 2004). Beyond taxanes, XIAP has been shown to confer resistance to Doxorubicin in breast cancer (Delbue et al., 2020), and to cisplatin in colon, ovarian, lung, and pancreatic cancers (Xiong et al., 2017; Xu et al., 2017; Ma et al., 2009; Lopes et al., 2007). Additionally, XIAP is implicated in resistance to Gemcitabine in pancreatic cancer (Shrikhande et al., 2006), and to Carboplatin in ovarian cancer (Thibault et al., 2018). XIAP also contributes to resistance against Vincristine in neuroblastoma (Frommann et al., 2018). Furthermore, XIAP induces resistance to tyrosine kinase inhibitors such as Lapatinib in breast cancer (Aird et al., 2010), and Imatinib in acute myeloid leukemia (AML) (Silva et al., 2013). By directly inhibiting caspases and blocking apoptosis, XIAP allows cancer cells to evade cell death mechanisms triggered by these drugs, leading to treatment failure and disease progression.

Survivin, as summarized in Table 1, is associated with resistance to Sorafenib and Doxorubicin in hepatocellular carcinoma (HCC) (Sun et al., 2021; Liu et al., 2023). It also contributes to resistance to Doxorubicin in breast cancer and AML (Wang et al., 2003), as well as Vincristine in lung cancer, acute lymphoblastic leukemia (ALL), and myeloma (Zhou J. et al., 2018; Park et al., 2011; Tsubaki et al., 2015). Furthermore, survivin induces resistance to taxanes such as Docetaxel in lung cancer and Taxol in ovarian and lung cancers (Zaffaroni et al., 2002). Resistance to DNA-damaging agents is also evident, with survivin implicated in resistance to Cisplatin in bladder (Krafft et al., 2019), gastric (Dong et al., 2014; Sun et al., 2014), and lung cancers (Hu et al., 2016), and Cytarabine in AML (Stroopinsky et al., 2018). It also mediates resistance to Gemcitabine in pancreatic cancer (Fuller et al., 2022) and Daunorubicin in ALL (Wu et al., 2008). Additionally, survivin reduces the effectiveness of targeted therapies, such as Imatinib in chronic myeloid leukemia (CML) (Speletas et al., 2011; Carter et al., 2006) and Gefitinib in lung cancer (Zhou et al., 2018).

By interfering with apoptosis and promoting treatment resistance, XIAP and survivin represent two critical therapeutic targets for overcoming drug resistance and improving cancer treatment outcomes, as discussed in Section 3. Furthermore, IAPs also contribute to resistance to immunotherapies (Evans et al., 2016; Straszewski-Chavez et al., 2004; Sugihara et al., 2020), radiation (Holcik et al., 2000; Sun et al., 2011), TRAIL (tumor necrosis factor-related apoptosis-inducing ligand) (Gillissen et al., 2013; Ndozangue-Touriguine et al., 2008), suggesting that their pivotal role and they may serve as a vulnerability to reverse drug resistance.

## 3 Targeting IAP to overcome drug resistance in cancers

**FIGURE 2:**
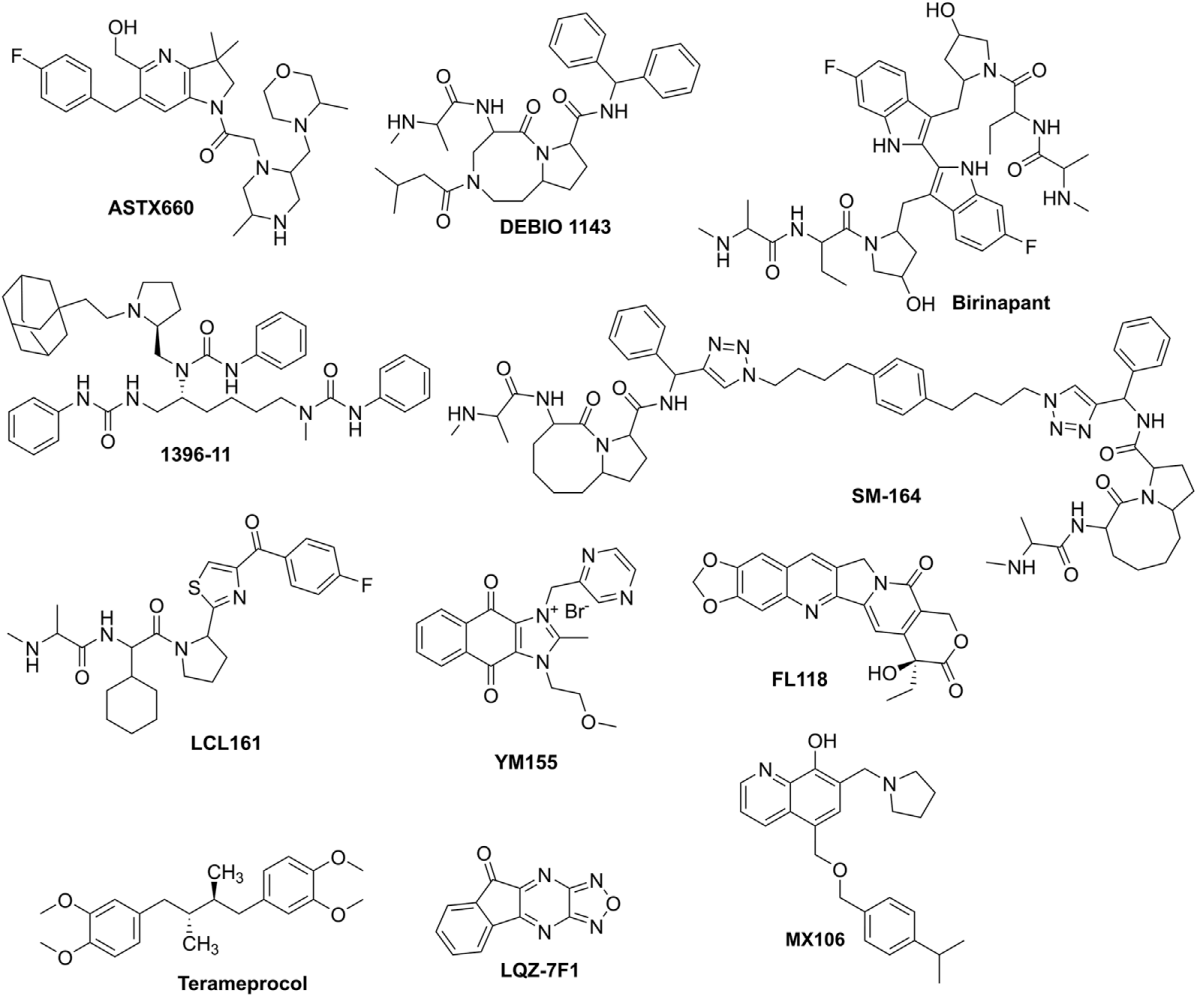
The prominent IAPs that have been used for combination to combat drug resistance in cancers.

Therapeutic strategies targeting IAPs focus on antagonizing their antiapoptotic activity, restoring caspase function, and sensitizing cancer cells to treatment. In this review, we took a spot on small-molecule inhibitors. To clarify, non-specific inhibitors, such as many compounds derived from natural products, are excluded from this review. These small-molecule inhibitors (listed in Table 2; Figure 2), including 1) c-IAP1/2 inhibitors such as ASTX660, birinapant, and DEBIO 1143, which are all under clinical trials; 2) XIAP inhibitors, such as drug candidate SM-164, and LCL161; and 3) survivin inhibitors which disrupt its role in apoptosis inhibition and mitosis regulation. Examples include drug candidates YM155, FL118, and Terameprocol, etc. Other compounds in laboratory or preclinical studies are also included. It should be noted that, since IAPs share similar 3D motifs in their structures, thus, many of these small-molecule inhibitors may be able to target and bind to several IAPs. Furthermore, due to similar reasons, those IAPs inhibitors have demonstrated overlapping yet slightly different mechanisms, i.e., reversing IAPs-mediated caspases activation, thereby inducing apoptosis of intrinsic or extrinsic when combined with other conventional or targeted therapies.

**TABLE 2:** Summary of using IAP Inhibitors to overcome drug resistance in cancers.

IAP	Inhibitors^a^	Combinations	Cancer types	References
cIAP1/2	ASTX660^b^	Cisplatin	Cervical cancer	Hoskin et al. (2024)
	DEBIO 1143	Carboplatin	Ovarian cancer	Thibault et al. (2018)
		Cisplatin	Head and neck cancer	Sun et al. (2020), Le Tourneau et al. (2020)
		Docetaxel	Lung cancer	Langdon et al. (2015)
		Venetoclax	Colorectal cancer	Perimenis et al. (2016)
	Birinapant	Venetoclax	Colorectal cancer	Perimenis et al. (2016)
		Gemcitabine	TNBC	Xie et al. (2021)
		Taxol	TNBC	Shu et al. (2019)
			Pancreatic cancer	Wang W. et al. (2018)
		Ralimetinib	NSCLC	Colombo et al. (2020)
		Bortezomib	Myeloma	Zhou et al. (2019)
		Carboplatin	Ovarian cancer	Singh et al. (2022)
		5-Azacytidine	AML	Carter et al. (2014)
		Dacarbazine	Melanoma	Vetma et al. (2017)
		Bazedoxifene	Colorectal cancer	Dmello et al. (2024)
XIAP	1,396–11	Vinorelbine/cisplatin	NSCLC	Dean et al. (2010)
	SM-164	Doxorubicin	Osteosarcoma	Chen et al. (2019)
			HCC	Zhang et al. (2012)
		Gemcitabine	Pancreatic cancer	Zhou et al. (2013)
	LCL161^c^	Vincristine	Neuroblastoma	Langemann et al. (2017), Frommann et al. (2018)
		Auranofin	ALL	Hass et al. (2016)
		Bazedoxifene	Colorectal cancer	Dmello et al. (2024)
		Panobinostat	Myeloma	Zhou et al. (2021)
		Taxol	NSCLC	Yang et al. (2016)
			HCC	Tian et al. (2014)
			TNBC	Bardia et al. (2018)
		Vincristine	Neuroblastoma	Langemann et al. (2017)
		Gemcitabine/cisplatin	Cholangiocarcinoma	Prasopporn et al. (2022)
		Navitoclax	Breast cancer	Lee et al. (2020)
		Vincristine/cisplatin	Medulloblastoma	Chen et al. (2016)
		5-Azacytidine	ALL	Gerges et al. (2016)
Survivin	YM155	Cisplatin	Malignant rhabdoid tumor	Coyle et al. (2022)
			Head and neck cancer	Kumar et al. (2012)
			Osteosarcoma	Gao et al. (2015)
		Cabazitaxel	Prostate cancer	Miyao et al. (2020)
		Doxorubicin	Osteosarcoma	Zhang et al. (2015)
		Erlotinib	Lung cancer	Okamoto et al. (2012), Shimizu et al. (2020)
		Taxol	Lung cancer	Baspinar et al. (2019)
		Taxol/carboplatin	NSCLC	Kelly et al. (2013)
		Docetaxel	TNBC	Kaneko et al. (2013)
		Decitabine	AML	Yao et al. (2024)
		Gemcitabine	Pancreatic cancer	Yoon et al. (2012)
		Rapamycin	Renal cancer	Koike et al. (2014)
		ABT-737	Renal cancer	Woo et al. (2017)
		Rituximab	Non-Hodgkin lymphoma	Papadopoulos et al. (2016)
	FL118	Irinotecan/topotecan	Colon cancer	Ling et al. (2015)
		Irinotecan	Colorectal cancer	Kim et al. (2024)
		Gemcitabine	Pancreatic cancer	Ling et al. (2018)
	Terameprocol	Everolimus	Endometrial cancer	Chao et al. (2018)
		Temozolomide	Glioblastoma	Kimura et al. (2023)
	LQZ-7F1	Docetaxel	Prostate cancer	Peery et al. (2022)
	MX106	Doxorubicin	Breast cancer	Wang X. et al. (2018)

^a^ Antagonists are also included in this table.

^b^ ASTX660 is also an antagonist targeting XIAP.

^c^ LCL161 also targets c-IAP2.

Note: NSCLC, non-small cell lung cancer; TNBC, triple-negative breast cancer; CML, chronic myeloid leukemia; CLL, chronic lymphocytic leukemia; SCLC, small cell lung cancer; HCC, hepatocellular carcinoma; AML, acute myeloid leukemia; ALL, acute lymphoblastic leukemia.

DEBIO 1143 is an inhibitor used in combination with other drugs to overcome drug resistance in cancers, as summarized in Table 2. Specifically, DEBIO 1143 is combined with Carboplatin for the treatment of ovarian cancer (Thibault et al., 2018). Additionally, it is used with Cisplatin for head and neck cancer in phase 1 and 2 clinical trials, which showed promising effects among high-risk locoregionally advanced patients (Sun et al., 2020; Le Tourneau et al., 2020).

Birinapant is an IAP inhibitor used in various combinations to combat drug resistance in different cancers, as detailed in Table 2. It is combined with Venetoclax (Perimenis et al., 2016) and Bazedoxifene for colorectal cancer (Dmello et al., 2024). For triple-negative breast cancer (TNBC), Birinapant is used with Gemcitabine (Xie et al., 2021) and Taxol (Shu et al., 2019). It is also paired with Ralimetinib for non-small cell lung cancer (NSCLC) (Colombo et al., 2020), and with Bortezomib for myeloma (Zhou et al., 2019). Additionally, Birinapant is combined with Carboplatin for ovarian cancer (Singh et al., 2022), 5-Azacytidine for AML (Carter et al., 2014), Dacarbazine for melanoma (Vetma et al., 2017). This showcases Birinapant’s versatility in enhancing treatment efficacy across multiple cancer types when used in combination therapies. While extensive clinical trials have been conducted, it has been inactive recently for cancer treatment.

SM-164 is an IAP inhibitor that has been studied in combination with various drugs to overcome drug resistance in different cancers, as shown in Table 2. It is combined with Doxorubicin to treat osteosarcoma (Chen et al., 2019) and HCC (Zhang et al., 2012). Additionally, SM-164 is used with Gemcitabine for pancreatic cancer (Zhou et al., 2013). Currently, no active clinical trials have been scheduled.

LCL161, another IAP inhibitor, is utilized in combination therapies for various cancers. It is combined with Vincristine for neuroblastoma (Langemann et al., 2017; Frommann et al., 2018), Auranofin for ALL (Hass et al., 2016), and Panobinostat for myeloma (Zhou et al., 2021). LCL161 is also combined with Taxol for NSCLC (Yang et al., 2016), HCC (Tian et al., 2014), and TNBC (Bardia et al., 2018), and with Gemcitabine/cisplatin for cholangiocarcinoma (Prasopporn et al., 2022). Furthermore, it is used with Navitoclax for breast cancer (Lee et al., 2020), Vincristine/cisplatin for medulloblastoma (Chen et al., 2016), and 5-Azacytidine for AML (Gerges et al., 2016), demonstrating its wide application in improving treatment outcomes across diverse cancer types. LCL161 has been extensively tested in clinical trials for cancers in combination; however, it has not been approved yet.

YM155, a drug candidate, is a small-molecule inhibitor specifically designed to target the transcription factor survivin, thereby promoting cell survival and inhibiting apoptosis. YM155 works by suppressing the expression of survivin, which can potentially enhance the sensitivity of chemotherapeutics. In clinical and preclinical studies, YM155 has been combined with various chemotherapeutic agents to enhance treatment efficacy across various cancers. YM155 has been combined with Cisplatin, showing promising results in reducing tumor growth in malignant rhabdoid tumor, an aggressive pediatric cancer (Coyle et al., 2022). Combined with Cisplatin, YM155 has demonstrated synergistic effects in reducing cancer cell viability of head and neck cancers (Kumar et al., 2012). Two studies have highlighted the use of YM155 with Doxorubicin. One study focused on the combination’s effect on reducing tumor burden of osteosarcoma (Gao et al., 2015), while another explored its role in inducing apoptosis in osteosarcoma cells (Zhang et al., 2015). When combined with Cabazitaxel, YM155 has shown to enhance the cytotoxic effects of the chemotherapy, potentially offering a more effective treatment for advanced prostate cancer (Miyao et al., 2020). YM155 has been paired with Erlotinib, a targeted therapy for lung cancer, showing improved outcomes by targeting different pathways of cancer cell survival (Okamoto et al., 2012; Shimizu et al., 2020). Additionally, Taxol has been combined with YM155, indicating potential benefits in NSCLC treatment (Baspinar et al., 2019). For NSCLC specifically, the combination of Taxol and Carboplatin with YM155 has been researched, with some evidence of increased efficacy (Kelly et al., 2013). Docetaxel combined with YM155 has been studied for TNBC, showing potential in reducing cancer cell proliferation (Kaneko et al., 2013). Decitabine, a DNA hypomethylating agent, has been combined with YM155 for AML, aiming to increase the sensitivity of leukemia cells to treatment by altering gene expression patterns (Yao et al., 2024). Gemcitabine, a standard chemotherapy for pancreatic cancer, when used with YM155, has shown to potentially improve patient outcomes by targeting different aspects of cancer cell survival mechanisms (Yoon et al., 2012). Two different combinations have been explored for renal cancer; Rapamycin (Koike et al., 2014), which inhibits mTOR, and ABT-737 (Woo et al., 2017), a BH3 mimetic, both when used with YM155, have shown synergistic effects in promoting cancer cell death. Rituximab, a monoclonal antibody, has been combined with YM155 to target B-cell lymphomas, enhancing the immunological and apoptotic effects on lymphoma cells (Papadopoulos et al., 2016). However, no active clinical trials for YM155 are now undergoing. Interestingly, research showed that c-IAP1 overexpression could induce resistance to YM155 (Jung et al., 2015), suggesting that the members of IAPs have compensatory effects.

## 4 Future perspectives

The above information has validated that targeting IAPs may have high potential in overcoming MDR in cancer. However, challenges remain. Here, we attempted to predict the potential future directions.

(1) The development of specific IAP inhibitors, including proteolysis targeting chimeras (PROTACs). Inhibitors that specifically target IAPs are crucial for drug development. Traditional inhibitors might lack precision, leading to side effects or resistance. A novel approach involves using PROTACs, which degrade IAPs rather than just blocking them, potentially offering a more definitive way to eliminate these proteins from cancer cells.
(2) Exploring more combination therapies. Combining IAP-targeted treatments with other cancer therapies like immunotherapy, targeted treatments could enhance their effectiveness. Such combinations could disrupt multiple survival pathways in cancer cells, reducing their ability to adapt and survive. Exploring more effective combinations may potentially lead to customized treatment strategies based on the unique characteristics of each patient.
(3) Biomarker discovery. Identifying biomarkers that can predict patient response to IAP-targeted treatments is essential for personalized medicine. This would involve looking for signs of IAP activity or related pathways within tumors. Advanced technologies for analyzing genetics, proteins, and even tumor metabolism are key here. Discovering these biomarkers would help in selecting the right patients for clinical trials, improving the chances of successful outcomes.
(4) Extensive clinical trials. To move IAP-targeted therapies from bench-side to clinical use, comprehensive trials are needed. These should evaluate not just their safety and effectiveness but also the best ways to administer them, including dosage and combination with other treatments. Long-term studies are also important to monitor sustained effectiveness, potential long-term side effects, and resistance development.

## 5 Conclusion

IAPs have been validated in inducing drug resistance and MDR in various cancers. Growing studies have shown that targeting IAPs is a feasible approach to overcome drug resistance in cancers. By restoring apoptotic pathways and disrupting survival mechanisms, IAPs inhibitors have the potential to improve outcomes for patients with drug-resistant cancers significantly.
